# *HOXC4* up-regulates NF-κB signaling and promotes the cell proliferation to drive development of human hematopoiesis, especially CD43+ cells

**DOI:** 10.1097/BS9.0000000000000054

**Published:** 2020-09-01

**Authors:** Jiahui Zeng, Wencui Sun, Jing Chang, Danying Yi, Lijiao Zhu, Yonggang Zhang, Xu Pan, Ya Zhou, Mowen Lai, Guohui Bian, Qiongxiu Zhou, Jiaxin Liu, Bo Chen, Feng Ma

**Affiliations:** a Institute of Blood Transfusion, Chinese Academy of Medical Sciences & Peking Union Medical College (CAMS & PUMC), Chengdu 610052, China; b State Key Laboratory of Biotherapy, Sichuan University, Chengdu 61006, China; c State Key Laboratory of Experimental Hematology, CAMS & PUMC, Tianjin 300020, China

**Keywords:** CD43, Erythroid–megakaryocyte common progenitor (EMkP), Hematopoiesis, *HOXC4*, Human embryonic stem cells (hESCs), Inducible expression

## Abstract

The hematopoietic function of *HOXC4* has not been extensively investigated. Our research indicated that induction of *HOXC4* in co-culture system from D10 significantly promoted productions of most hematopoietic progenitor cells. CD34−CD43+ cells could be clearly classified into CD34−CD43^low^ and CD34−CD43^high^ sub-populations at D14. The former cells had greater myelogenic potential, and their production was not significantly influenced by induction of *HOXC4*. By contrast, the latter cells had greater potential to differentiate into megakaryocytes and erythroid cells, and thus had properties of erythroid–megakaryocyte common progenitors, which abundance was increased by ∼2-fold when *HOXC4* was induced from D10. For CD34−CD43^low^, CD34+CD43+, and CD34−CD43^high^ sub-populations, CD43 level served as a natural index for the tendency to undergo hematopoiesis. Induction of *HOXC4* from D10 caused more CD43+ cells sustain in S-phase with up-regulation of NF-κB signaling, which could be counteracted by inhibition of NF-κB signaling. These observations suggested that promotion of hematopoiesis by *HOXC4* is closely related to NF-κB signaling and a change in cell-cycle status, which containing potential of clinical applications.

## 1 INTRODUCTION

The class I homeobox (HOX) family of homeodomain-containing transcription factors has 39 members in mammals. These genes are distributed throughout the genome in four clusters: HOXA (7p15), HOXB (17q21), HOXC (12q13), and HOXD (2q31).^1,2^ These genes exhibit high conservation and redundancy of function.^3^

*HOXC4* is actively transcribed in human CD34+/CD38^low^ and CD34+/CD38+ progenitors, as well as during the development and differentiation of lymphoid, myeloid, and erythroid lineages.^4^ HOXC4 protein can be detected in proliferating lymphocytes, but not in resting lymphocytes, even though the transcript is present in the latter cells, indicating that its production is regulated post-transcriptionally.^5^ The high expression of HOXC4 protein in proliferating hematopoietic cells suggests that it plays a role in maintaining proliferation. As with *HOXB4*, overexpression of *HOXC4* in stromal cells or hematopoietic stem cells (HSC) promotes expansion and production of hematopoietic cells, and improves the engraftment efficiency of HSCs,^6–8^ raising the possibility of clinical applications in regenerative medicine. However, the roles of *HOXC4* enacted during the various stages of hematopoiesis have not been explored in detail or elucidated in a model organism or hESC-based in vitro system.

A tet-on system based on *piggy*Bac with a green fluorescent protein (GFP) tag, PB-Tet-on-OE, has been developed and successfully used to establish transgenic hESC lines in our center.^9,10^ This reliable gene delivery system allows manipulation and tracking of the expression of target genes during mesoderm induction and hematopoiesis originating from hESCs with a much higher resolution and lower background than the traditional lentiviral system.^9,11^ Moreover, it has the potential to be adapted to a novel inducible expression system that controls the expression modes of two or more foreign genes.^12^ The aorta–gonad–mesonephros-derived stromal cell (AGM-S3) co-culture system mimics definitive hematopoiesis to some degree. It is a useful in vitro system for observing the function of key genes during normal or abnormal hematopoiesis, including *HOXC4*, and has potential utility in screening for compounds that promote human hematopoiesis.^13^

Recently, new conceptions of HSC-independent and HSC-dependent hematopoiesis routes have been proposed, and these models have become widely accepted.^14^ The classically defined hematopoietic populations need to be reconsidered according to these conceptions, as the detailed properties of these two parallel routes of hematopoiesis remain unclear. In this study, using the AGM-S3 co-culture system, we dissected the function of *HOXC4* on hematopoiesis according to these new views. Our preliminary results from in vitro system provide clear evidence that overexpression of *HOXC4* could promote hematopoiesis via both routes, and that its effects are closely related to the protein level of CD43 and NF-κB signaling, suggesting a possible molecular and cellular mechanism for the function of *HOXC4* during human hematopoiesis.

## 2 RESULTS

### 2.1 *HOXB4* is expressed in a different pattern in co-cultured H1 hESCs than *HOXA4/HOXC4/HOXD4*, and shares low sequence similarity with the other genes

We monitored mRNA expression of *HOXA4/HOXB4/HOXC4/HOXD4* at various times in H1 hESCs co-cultured with AGM-S3. Expression of all four genes gradually decreased during hematopoietic development, and reached a minimum at D10 (Fig. S1). *HOXA4/HOXC4/HOXD4* were more highly expressed during mesoderm induction (D0–D4) than during hematopoietic differentiation (D4–D14), although a significant increase was observed at D14 for *HOXA4*. *HOXB4* was expressed differently during hematopoiesis than the other three members of the *HOX* family, including *HOXC4*. Amino acid sequence alignment revealed low sequence similarity between HOXB4 and HOXA4/HOXC4/HOXD4 (Fig. S2), indicating that these proteins might serve different functions during hematopoiesis.

### 2.2 Transgenic hESCs exhibit normal inducible expression and pluripotency

**Figure 1:**
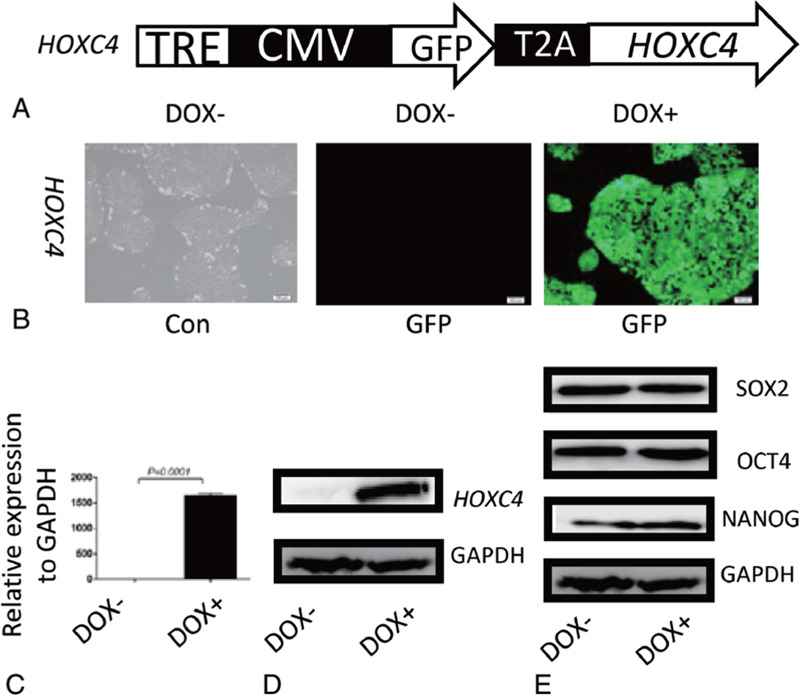
Induction and pluripotency of *HOXC4*-inducible transgenic hESC lines. (A) Schematic representation of the *piggy*Bac constructs used to express *HOXC4*. CMV mini, cytomegalovirus minimum promoter; TRE, tet-on regulation element; T2A, Thosea asigna virus 2A peptide. After *HOXC4*/hESCs were induced by DOX for 48 h, (B) the cells were photographed under a fluorescence microscope, and co-expression of GFP was observed. At the same time, (C) qRT-PCR detection was performed to confirm that inducible expression of *HOXC4* was highly stringent and efficient at the transcriptional level, and (D) western blotting detection was performed to confirm *HOXC4* overexpression at protein level. (E) The pluripotency of *HOXC4*/hESCs was confirmed by western blotting for SOX2, OCT4, and NANOG.

We constructed PB-Tet-on-GFP-T2A-h*HOXC4* and established the corresponding H1-derived inducible *HOXC4*/hESC line (Fig. 1A). Monitoring of *HOXC4*/hESCs treated with or without DOX for 48 hours by fluorescence microscopy (Fig. 1B), qRT-PCR (Fig. 1C), and western blotting assays (Fig. 1D) revealed highly stringent and efficient induction of *HOXC4*. Pluripotency of induced or non-induced *HOXC4*/hESCs was confirmed by western blotting to detect SOX2, OCT4, and NANOG proteins (Fig. 1E).

### 2.3 *HOXC4* overexpression from D6 or later broadly promotes hESC-derived hematopoiesis

**Figure 2:**
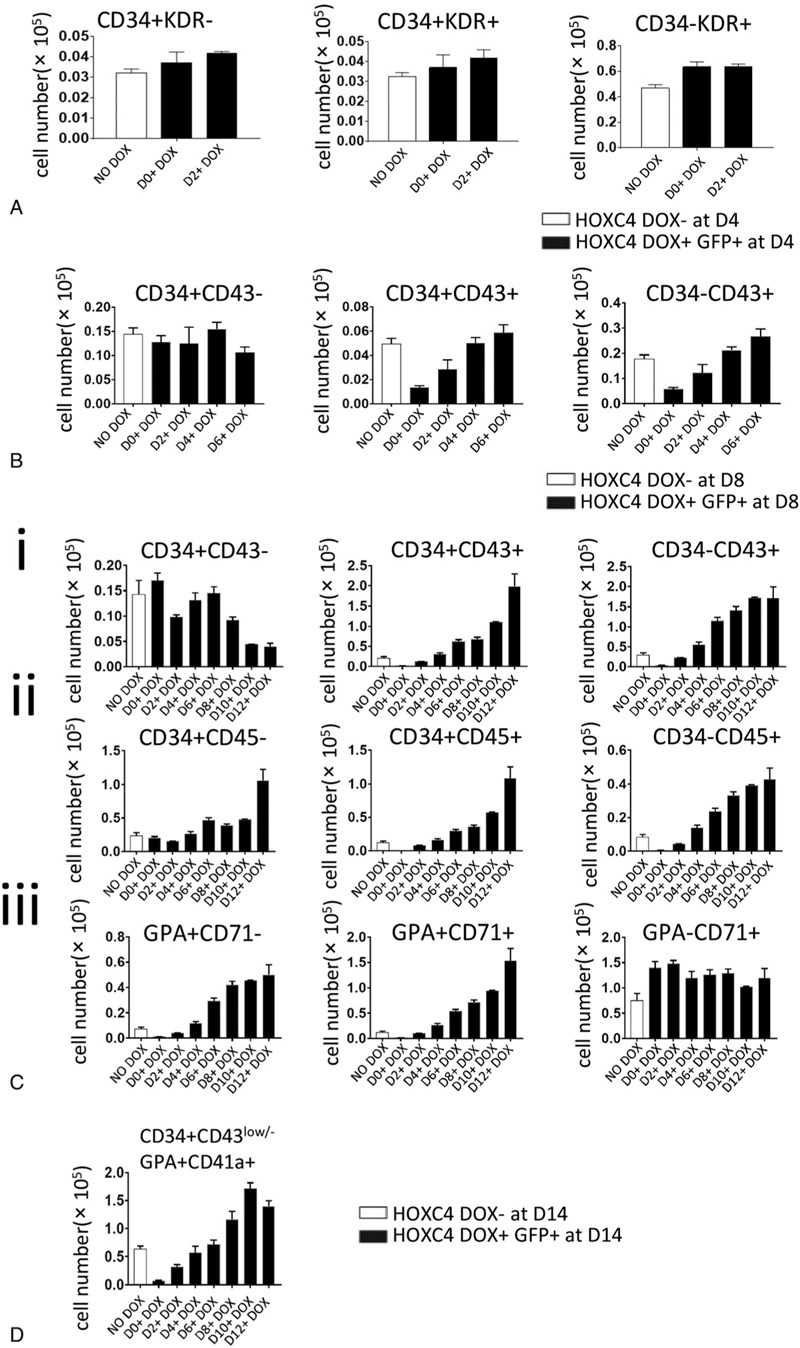
Ectopic expression of *HOXC4* from D6 promoted hematopoiesis. *HOXC4*/hESCs co-cultured with AGM-S3 cells were treated with DOX from D0, D2, D4, D6, D8, D10, or D12, and with FACS using the indicated combination of antibodies against (A) KDR/CD34 (at D4), (B) CD34/CD43 (at D8), (C) GPA/CD71, CD34/CD43, and CD34/CD45 (at D14), and (D) CD34/CD43/GPA/CD41a (at D14). The results were compared between non-induced co-cultures and the GFP+ fraction of co-cultures treated with DOX from D0, D2, D4, D6, D8, D10, or D12. Production of CD34+CD43+, CD34−CD43+, CD34−CD45+, CD34+CD45+, GPA+CD71+, and GPA+CD71− populations at D14 was dramatically increased by *HOXC4* induced from D6 or later, indicating that *HOXC4* has a strong positive effect on hematopoiesis. The EMkP-like population (CD34^low^/^−^CD43^high^GPA+CD41a+) at D14 were dramatically increased by induction of *HOXC4* from D6 or later, indicating a strong positive effect of *HOXC4* on this population.

*HOXC4*/hESCs co-cultured with AGM-S3 cells were induced to overexpress *HOXC4* during different stages of hematopoiesis and then were subjected to FACS at D4, D8, and D14. Observations at D14 indicated that *HOXC4* overexpression broadly promoted the production of hematopoietic populations, including CD34+CD43+, CD34−CD43+, CD34+CD45+, CD34−CD45+, GPA+CD71+, and particularly, CD34−CD43+ cells (Fig. 2C; Fig. S3C). By contrast, no significant effects on hematopoietic populations, such as CD34+CD43+ and CD34−CD43+, were observed at D8 (Fig. 2B; Fig. S3B). Together, these findings indicate that hematopoiesis at D14 was significantly promoted by overexpression of *HOXC4* from D6, and especially from D10. Severe inhibitory effects on CD34+CD43+ and CD34−CD43+ populations, but not on CD34+CD43− populations, were observed at D8 when induction began at D0 (Fig. 2B). Earlier detection (D4) revealed no obvious inhibitory effects on the KDR+CD34−, KDR+CD34+, and KDR−CD34+ populations (Fig. 2A; Fig. S3A). Thus, overexpression of *HOXC4* might only influence the production of hematopoietic cells, but not influence the induction of mesoderm and endothelium.

**Figure 3:**
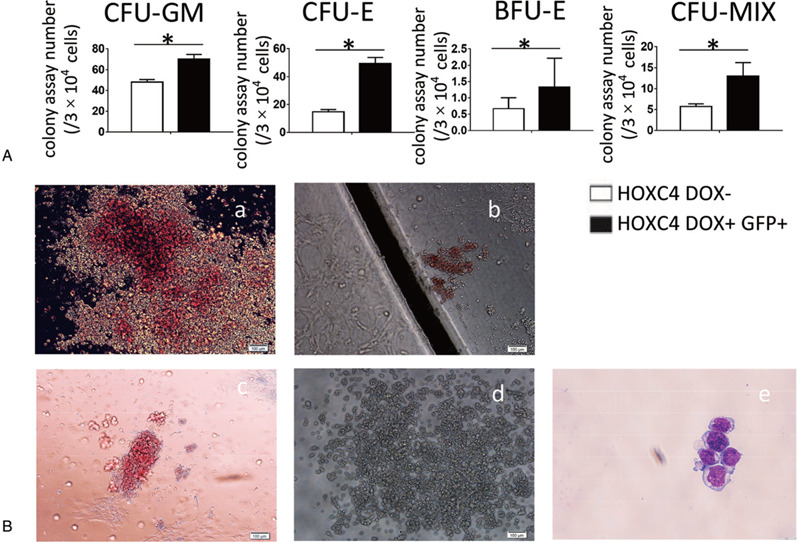
Overexpression of *HOXC4* from D10 increases both the erythrogenic and myelogenic potentials of co-cultured cells. The non-induced D14 co-culture of *HOXC4*/hESCs, or the GFP+ fraction of D14 co-culture of *HOXC4*/hESCs induced from D10 were sorted and subjected to colony assays to determine their hematopoietic potentials. (A) Colony number of each type of colony derived from 2 × 10^4^ co-cultured cells. *P* < .05 was considered significant. (B) Typical morphologies of CFU-Mix (a), CFU-E (b), BFU-E (c), and CFU-GM (d) colonies. Scale bar, 100 μm. MGG staining of cells in BFU-E colonies (e). Scale bar, 10 μm. Both myelogenic and erythrogenic potentials were significantly stimulated.

To clarify how the hematopoietic potentials of these populations were promoted by *HOXC4* overexpression, the GFP+ fraction or non-induced cells were sorted from the D10-induced or non-induced co-culture cells at D14, and cultured to form colonies. Colony numbers (colony-forming unit-granulocyte/macrophage (CFU-GM), colony-forming unit-erythroid (CFU-E), burst-forming unit-erythroid (BFU-E), colony-forming unit-mixed (CFU-Mix)) were significantly higher for induced cells than non-induced cells, indicating that DOX treatment increased hematopoietic potential (Fig. 3A). Typical colonies of CFU-GM, CFU-E, BFU-E, and CFU-GM were observed (Fig. 3B,a–d), and erythroid cells were confirmed by May–Grunwald–Giemsa staining (MGG) (Fig. 3B,e).

### 2.4 *HOXC4* promotes hematopoiesis originating from HSC-independent routes

The FACS analysis at D14 revealed that both erythroid–megakaryocyte common progenitors (EMkP)-like cells (CD34^low/−^CD43+GPA+CD41a+), the classic populations of HSC-independent, were more abundant when the cultures were treated with DOX from D6, especially from D10, indicating that overexpression of *HOXC4* can promote hematopoiesis originating from HSC-independent routes (Fig. 2D; Fig. S5).

### 2.5 *HOXC4* significantly promotes the production of CD43+ cells and the potential of its sub-populations

**Figure 4:**
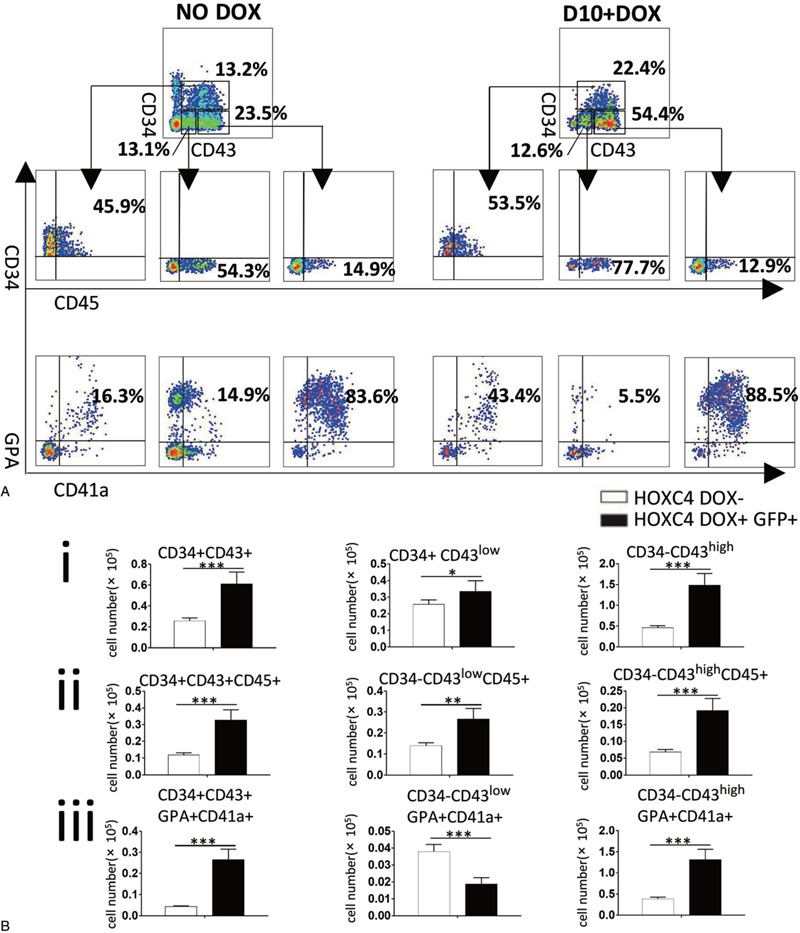
Sub-populations of CD43+ cells have different components, and their production is promoted by induction of *HOXC4*. (A) *HOXC4*/hESCs co-cultured with AGM-S3 were induced to overexpress *HOXC4* from D10, and were subjected to FACS analysis at D14. CD34+CD43+ sub-populations mainly contained CD34+CD45+, CD34−CD43^low^ mainly contained CD45+ cells; and CD34−CD43^high^ contained a high proportion of GPA+CD41a+ cells. (B) Production of these three sub-populations and of the hematopoietic populations they contained was promoted by treatment with DOX, indicating that overexpression of *HOXC4* from D10 strongly promoted hematopoiesis from CD43+ cells.

FACS detection at D14 revealed that three sub-populations of CD43+ cells, including CD34+CD43+, CD34−CD43^high^, and CD34−CD43^low^ cells, were promoted (About 2-, 3-, and 1.2-fold, respectively) by induction of *HOXC4* from D10. Production of CD45+ cells and GPA+CD41a+ cells in these sub-populations was significantly elevated following DOX treatment (except that the number of GPA+CD41a+ cells decreased in the CD34+CD43^low^ sub-population), indicating that overexpression of *HOXC4* from D10 strongly stimulates the production of myeloid progenitors and erythroid progenitors in most CD43+ cells (Fig. 4).

### 2.6 Sub-populations of CD43+ cells have different hematopoietic potential

**Figure 5:**
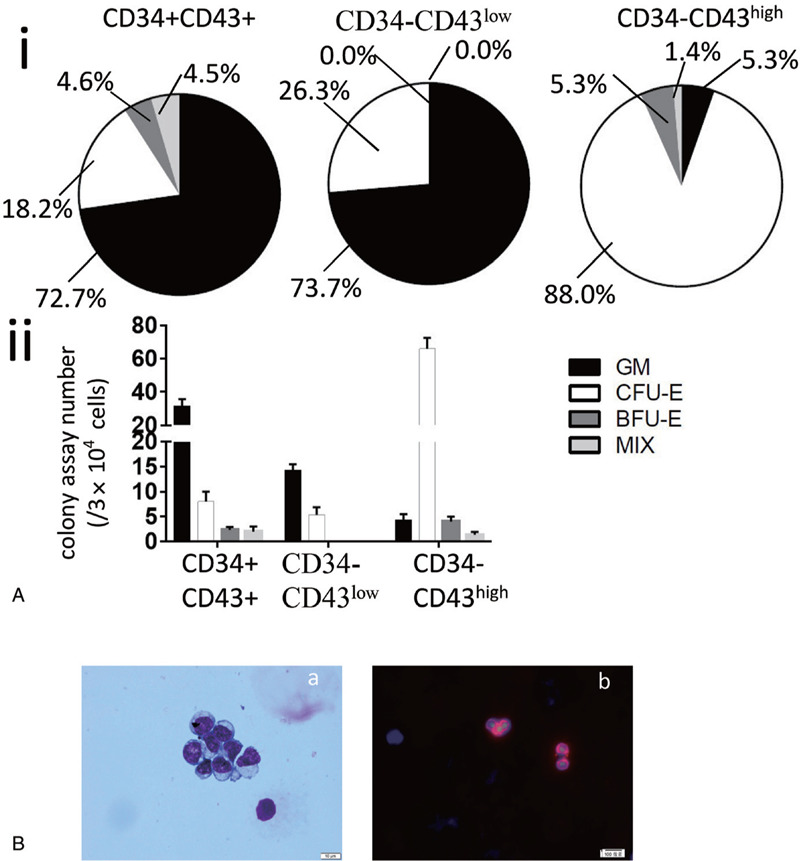
Sub-populations of CD43+ cells have different hematopoietic potential in colony culture assays. The CD34+CD43+, CD34−CD43^low^, and CD34−CD43^high^ sub-populations were sorted from D14 non-induced *HOXC4*/hESC co-cultures. (A) About 3 × 10^4^ sorted cells of each type were subjected to colony assays to determine their hematopoietic potential. (B) MGG staining of cells in BFU-E colonies (a), immunofluorescence staining of cells in BFU-E colonies (b). Colony culture assays indicated that CD34+CD43+ and CD34−CD43^low^ have a high myelogenic potential, whereas CD34−CD43^low^ have high erythrogenic potential.

Colony culture assays of CD34+CD43+, CD34−CD43^low^, and CD34−CD43^high^ sorted from non-induced co-cultures at D14 revealed that these sub-populations had distinct differentiation potentials. Colonies formed from CD34+CD43+ cells were mainly CFU-GM colonies, and also contained fewer CFU-E, CFU-MIX, and BFU-E colonies. CD34−CD43^low^ cells had the lowest overall hematopoietic potential, but the colonies they formed contained the highest proportion of CFU-GM colonies and a much lower proportion of CFU-E colonies; they lacked BFU-E and CFU-MIX colonies altogether. The colonies formed by CD34−CD43^high^ cells contained the highest proportion of CFU-E colonies and much lower proportions of CFU-GM, BFU-E, and CFU-MIX colonies (Fig. 5A). Classic BFU-E colonies were tested by MGG staining (Fig. 5B,a) and immunofluorescence staining (Fig. 5B,b) to confirm that they were erythroid cells.

### 2.7 *HOXC4* promoted expansion of myeloid progenitors and EMkP-like populations

**Figure 6:**
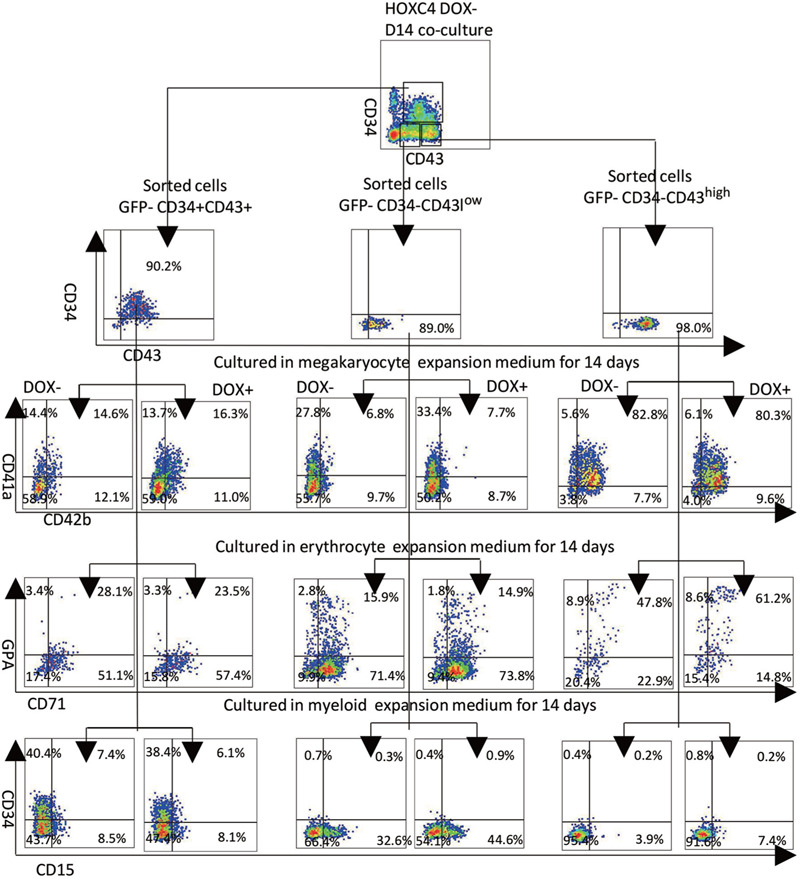
Sub-populations of CD43+ cells have different hematopoietic potentials in suspension culture assay, and differentiation was promoted by induction of *HOXC4*. The CD34+CD43+, CD34−CD43^low^, and CD34−CD43^high^ sub-populations were sorted from the corresponding co-culture cells. About 5 × 10^3^ sorted cells of each type were re-suspended in 250 μl myeloid, megakaryocyte, or erythroid expansion medium; seeded into each well of a 48-well plate to be further cultured with or without DOX; and subjected to FACS analysis at day 14. The suspension culture assays indicated that CD34−CD43^low^ had high myelogenic potential; CD34−CD43^high^ had high erythrogenic and plateletogenic potential (classic trait of EMkPs); and CD34+CD43+ had an intermediate phenotype.

CD34+CD43+, CD34−CD4^low^, and CD34−CD43^high^ sub-populations at D14 were further cultured in myeloid, megakaryocyte, or erythroid expansion medium (for 14, 7, or 14 days, respectively), with or without DOX. The results of flow analysis indicated that CD34+CD43+, CD34−CD43^low^, and CD34−CD43^high^ cells could differentiate into CD41a+CD42b+ cells (15%, 7%, and 82%, respectively), GPA+CD71+ cells (23%, 15.9%, and 61.2%, respectively), and CD34−CD15+ cells (7%, 44.6%, and 7.4%, respectively). Among them, CD34−CD43^high^ sub-populations had the highest differentiation potentials of megakaryocyte and erythroid, reflecting classic traits of EMkP-like populations, and *HOXC4* induction significantly promoted their erythroid potential. The CD34-CD43^low^ sub-population had the highest myeloid potential, which could be significantly increased by *HOXC4* induction. The differentiation potential of the CD34+CD43+ sub-population was intermediate between the two (Fig. 6).

### 2.8 The level of CD43 protein serves as an index of erythroid or myeloid differentiation potential

**Figure 7:**
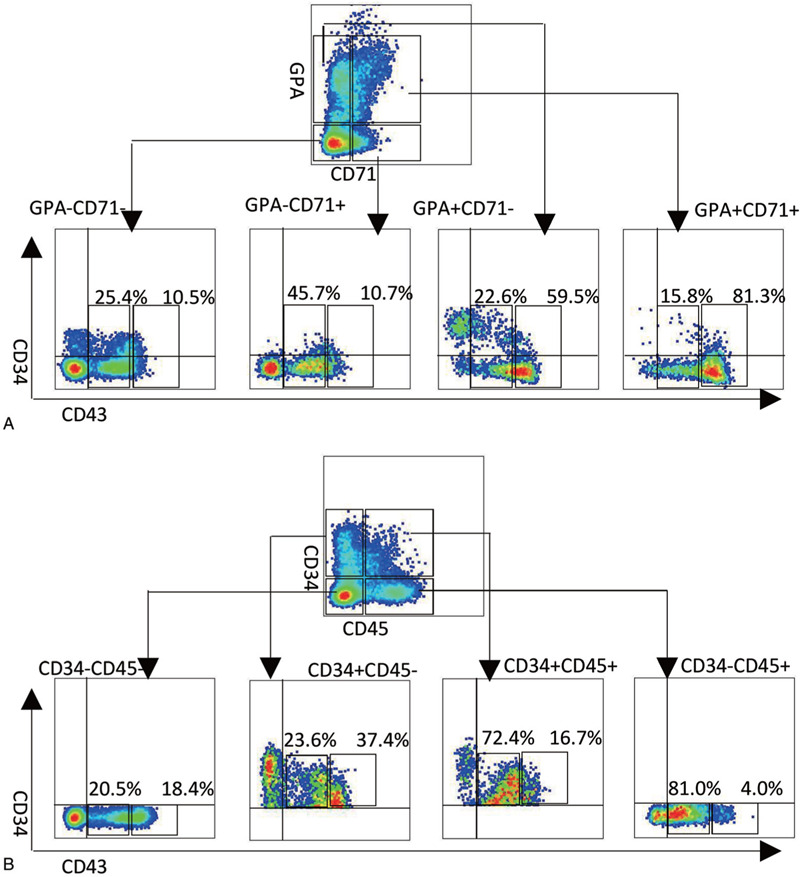
CD43 protein can be used as an index to distinguish the tendencies of hematopoietic differentiation potentials. Non-induced *HOXC4*/hESCs co-cultured with AGM-S3 were subjected to FACS analysis at D14 using the indicated combination of antibodies against (A) CD34/CD43/GPA/CD71 or (B) CD34/CD43/CD45. CD43^low^ cells had different proportions in CD34−CD45−, CD34+CD45−, CD34−CD45+, and CD34+CD45+ cells, and the ratios were positively related to their myelogenic potentials. CD43^high^ cells had different proportion in GPA−CD71−, GPA−CD71+, GPA+CD71−, and GPA+CD71+ cells, and the ratios were positively related to their erythrogenic potential.

GPA and CD45 at the late stage of hematopoiesis (eg, D14) are regarded as the main surface markers for erythroid and myeloid progenitors.^15–17^ FACS analysis indicated that GPA+ cells (especially GPA+CD71+ cells, as well as GPA+CD71− cells) contain a much higher proportion of CD43^high^ cells than GPA− (GPA−CD71+ and GPA−CD71− cells) cells (81.3% and 59.5% vs 10.7% and 10.5%) (Fig. 7A), whereas CD45+ cells (especially CD34−CD45+ cells, as well as CD34+CD45+ cells) contain a much higher proportion of CD43^low^ cells than CD45− (CD34+CD45− and CD34−CD45−) cells (81% and 72.4% vs 23.6% and 20.5%) (Fig. 7B). These results implied that at least in the AGM-S3 co-culture system, CD43^high^ is an index of erythrogenic potential, whereas CD43^low^ is an index of myelogenic potential, during hematopoiesis.

### 2.9 *HOXC4* overexpression up-regulates NF-κB signaling to promote hematopoiesis

**Figure 8:**
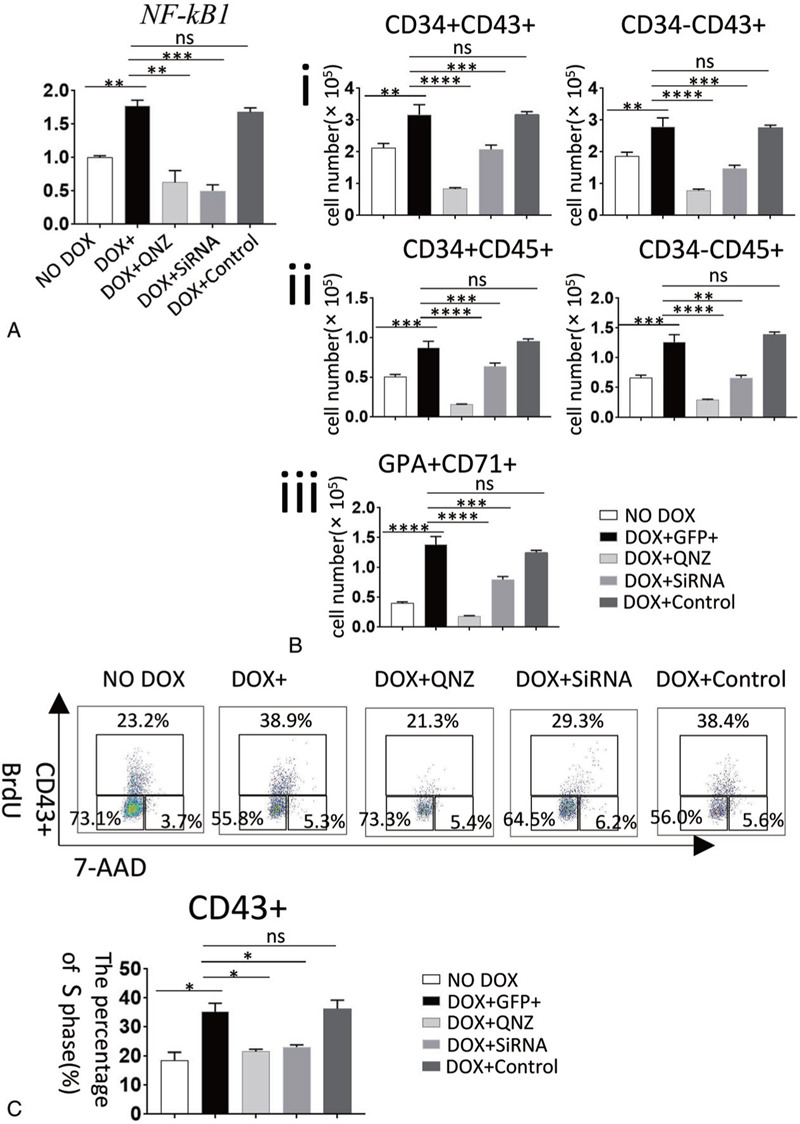
Promotion of hematopoiesis by *HOXC4* overexpression from D10 is closely related to NF-κB signaling and might be caused by a change in cell-cycle status. (A) qRT-PCR at D14 showed that *NF-κB1* was up-regulated when *HOXC4* was induced from D10, which could be counteracted by treatment with QNZ or *NF-κB1* siRNA. (B) If 20 nM QNZ or *NF-κB1* siRNA was added together with DOX from D10, FACS analysis at D14 showed that the positive effects of *HOXC4* overexpression on the CD34+CD43+, CD34−CD43+, CD34−CD45+, CD34+CD45+, and GPA+CD71+ populations were weakened or even abolished. (C) Analyses of cell-cycle status revealed that *HOXC4* induction from D10 increased the proportion of cells in S-phase in CD43+ cells of co-cultures, and that these effects were eliminated by treatment with QNZ or *NF-κB1* siRNA.

When *HOXC4*/hESCs co-cultured with AGM-S3 cells were induced from D10, qRT-PCR at D14 revealed that *NF-κB1* expression was up-regulated following induction which could be completely counteracted by addition of 20 nM QNZ (*NF-κB1* transcription inhibitor) or *NF-κB1* siRNA from D10 (Fig. 8A). In addition, cell-cycle assays revealed significant promotion of S-phase in CD43+ cells in co-cultures induced from D10. The positive effects of *HOXC4* on hematopoiesis and change in cell-cycle status were partially or completely counteracted by addition of 20 nM QNZ or *NF-κB1* siRNA (Fig. 8B–C, Fig. S4). This indicated that the increase in proliferation is the main cellular mechanism underlying the increased abundance of CD43+ cells, which is closely related to NF-κB signaling.

## 3 DISCUSSION

*HOX* genes that clustered at the same locus in the genome are likely to share similar traits, providing some key clues for studies of their functions.^18–20^ The group 4 *HOX* genes are *HOXA4*, *HOXB4*, *HOXC4*, and *HOXD.*^21–23^ Our qPCR analysis revealed distinctive profiles of mRNA expression among *HOXB4* and three other group members during hematopoiesis in H1 hESCs co-cultured with AGM-S3 cells (Fig. S1), and amino acid sequence alignment showed that except for the homeobox domains, the encoded proteins do not share a high degree of similarity (Fig. S2A), suggesting divergence of their functions. Among them, only the function of *HOXB4* has been intensively researched in the context of hematopoiesis.^24–26^ Previously, we showed that when *RUNX1b* is overexpressed at the early stage of mesoderm induction, hematopoiesis is strongly blocked,^9^ and many genes of the *HOX* family are down-regulated. *HOXC4* was one of the most down-regulated *HOX* genes (unpublished data). A highly conserved gene (Fig. S2B), *HOXC4* has positive effects on HSC expansion similar to those of *HOXB4*,^6,8^ but its function during human hematopoiesis has not been examined in an *in vitro* hematopoiesis system derived from hESCs.

In our experiments, *HOXC4*/hESCs co-cultured with AGM-S3 cells were induced to overexpress *HOXC4* on different days after the initiation of hematopoiesis, and were subsequently monitored by FACS analysis and colony culture assay. FACS analysis at D14 revealed that for multiple hematopoietic populations, including CD34+CD43+, CD34−CD43+, CD34+CD45+, CD34−CD45+, and GPA+CD71+, overexpression of *HOXC4* had similar influence at different stages. When induction began in the earliest stage (D0–D2), especially at D0, production was dramatically reduced at D14, whereas D4 KDR+ cells and D8 CD34+CD43− cells were not significantly affected (Fig. 2A–B; Fig. S3A–B), indicating that *HOXC4* induction from D0 can decrease production of the hematopoietic progenitors, but cannot influence induction of mesoderm or precursors of hematopoietic progenitors. When induced from D6 or later, production of all cell types was significantly increased, with effects growing larger when induction was started later (Fig. 2C). To facilitate detection, D10 was chosen as the day to start induction of *HOXC4*, which resulted in very strong positive effects on hematopoiesis, while at the same time yielding a higher ratio of GFP+ cells. Colony culture assays also demonstrated that colony numbers of CFU-GM, CFU-E, BFU-E, and CFU-MIX originating from D14 GFP+ co-cultures induced from D10 were significantly higher than in non-induced controls (Fig. 3). All results were consistent with a strong positive effect of *HOXC4* on hematopoiesis (including myelogenesis and erythrogenesis) at the late stage.

CD43+ cells include the main blood progenitors.^27–32^ In the AGM-S3 system, the CD43+ cells of the D14 co-culture could be obviously divided into three separated sub-populations: CD34+CD43+, CD34−CD43^high^, and CD34−CD43^low^. Their compositions of hematopoietic cells are distinct from each other. The CD34−CD43^high^ sub-population contained the highest proportion of GPA+CD41a+ cells (83.6%), whereas the CD34+CD43+ and CD34−CD43^low^ sub-populations contained much lower proportions of such cells (16.3% and 14.9%) (Fig. 4). The colony culture assay revealed that the CD34−CD43^high^ sub-population had very strong erythrogenic potential (Fig. 5). The results of further hematopoietic culture demonstrated that the CD34−CD43^high^ sub-population could produce a much higher proportion of erythroid and megakaryocyte progenitors, indicating that these cells have the classical traits of EMkP-like progenitors, with strong potentials for erythrogenesis and plateletogenesis (Fig. 6). The CD34+CD43+ and CD34−CD43^low^ sub-populations contained a higher proportion of CD45+ cells than the CD34−CD43^high^ sub-population (Fig. 4). They had similar CFU-GM numbers, whereas the CD34−CD43^low^ sub-population lacked CFU-E and CFU-MIX (Fig. 5A,i), and on the whole its colony-forming ability was much lower than that of the other two sub-populations (Fig. 5A,ii). The results of further hematopoietic culture demonstrated that the CD34−CD43^low^ sub-population could produce a much higher ratio of myeloid cells, indicating stronger myelogenic potential (Fig. 6).

Overexpression of *HOXC4* from D10 greatly increased the production of the CD34+CD43+ and CD34−CD43^high^ sub-populations, but not the CD34−CD43^low^ sub-population. The proportion of CD34+CD43+ cells that were GPA+CD41a+, and the proportion of CD34+CD43+ and CD34−CD43^low^ cells that were CD45+, were significantly increased by induction (Fig. 4A). Except for GPA+CD41a+ cells in the CD34−CD43^low^ sub-population, induction of *HOXC4* from D10 could promote production of GPA+CD41a+ and CD45+ cells in all three sub-populations (Fig. 4B). After the corresponding subsequent hematopoietic cultures, the erythrogenic potential of CD34−CD43^high^ and the myelogenic potential of CD34−CD43^low^ were significantly higher (Fig. 6). Together, these results indicated that *HOXC4* induction increases the production and potentials of all sub-populations of CD43+ cells in the late stage of co-culture, as well as in suspension cultures.

Now that the new conceptions of HSC-independent and HSC-dependent hematopoiesis routes have been widely accepted,^14^ it is reasonable to apply these models to evaluation of the hematopoietic populations produced from the AGM-S3 system. EMkPs develop via a route of hematopoiesis parallel to the one originating from HSCs.^33^ The CD34−CD43^high^ sub-population exhibited classic traits of EMkPs, which have strong potential for erythrogenesis and plateletogenesis, and might represent an HSC-independent population.^32,34–37^ On the other hand, CD34+CD43+ and CD34+CD45+ cells at D14 are a classic HSC-dependent population.^10,38,39^ Therefore, we tried to directly detect the effects of *HOXC4* induction on the representative populations of these two routes according to the classic definition of surface markers.^10,32,37,40–44^ Besides classic HSC-dependent populations, FACS also revealed that the induction of *HOXC4* from D10 significantly increased the production of EMkP-like progenitors (defined by CD34low/−CD43+GPA+CD41a+)^32^ (Fig. 2D; Fig. S5), which tendencies are nearly the same to other classic hematopoietic populations detected before (Fig. 2C). These findings demonstrated that *HOXC4* induction at the late stage has similar positive effects on hematopoiesis via both routes.

To elucidate the molecular/cellular mechanism of *HOXC4* on hematopoiesis, we checked the cell-cycle status of CD43+ cells and observed a significant increase in the proportion of cells in S-phase when *HOXC4* was induced from D10. At the same time, NF-κB signaling was up-regulated, and an inhibitor of NF-κB signaling, QNZ (*NF-κB1* transcription inhibitor), counteracted the positive effects on production and cell-cycle status. The results of corresponding RNA inference against *NF-κB1* also proved that the knockdown for key gene of NF-κB signaling had similar effects as its inhibitor did (Fig. 8). These observations indicate that activation of NF-κB signaling, stimulation of S-phase, and the positive effects of *HOXC4* on hematopoiesis (including myelogenesis and erythrogenesis) are closely related (Fig. 8). Moreover, this could explain how *HOXC4* promotes the proliferation of both HSC-dependent and -independent populations by up-regulating NF-κB signaling and accelerating their cell-cycle, with no obvious bias between these two routes or among any hematopoietic progenitors.

*HOXC4* induction in the late stage can significantly promote both routes of hematopoiesis, and the production of most key hematopoietic progenitors, by accelerating cell proliferation without obvious bias; this phenomenon is closely related to NF-κB signaling.^45^ In addition, the differentiation tendency toward erythrogenesis or myelogenesis, and the degree to which *HOXC4* promotes this tendency are strongly related to the expression level of CD43 protein in the target populations (Figs. 4 and 7). The hematopoietic population in that the higher CD43 protein was expressed could be stronger promoted production, and has stronger erythrogenesis potential but weaker myelogenesis (Fig. 4), which indicated that at least in the AGM-S3 system, the expression level of CD43 protein could serve as an index to assess the differentiation tendencies of target populations and the strength of the positive effect of *HOXC4* on these populations. The detailed molecular and cellular mechanism by which *HOXC4* promotes hematopoiesis still needs to be further explored, but it is clear that this knowledge will have clinical applications in the future.

## 4 MATERIALS AND METHODS

### 4.1 Establishment of a HOXC4-inducible transgenic hESC lines

The coding region of *HOXC4* was inserted between the *Swa*I and *Eco*RI sites of PB-Tet-on-OE to construct PB-Tet-on-GFP-T2A-h*HOXC4*, which was co-transfected into H1 hESCs along with the helper vector PB200PA-1 using Lipofectamine 3000 (Invitrogen, USA). Positive colonies were selected with 1 μg/ml puromycin, and then passaged using ReleSR (STEMCELL Technologies, Canada) to establish an inducible hESC line (*HOXC4*/hESCs) that co-expresses GFP. Induction by DOX was confirmed by quantitative reverse-transcription PCR (qRT-PCR) and western blot analyses. Pluripotency of induced or non-induced *HOXC4*/hESCs was confirmed by western blotting to detect SOX2, OCT4, and NANOG proteins.

### 4.2 Co-culture of hESCs with AGM-S3 cells

To induce hematopoietic differentiation, the H1 hESC line (generously provided by Prof. Tao Cheng) was co-cultured with AGM-S3 (a mouse stroma-derived cell line) as reported previously.^9,10^ This study was approved by the institutional ethics committee of the Institute of Blood Transfusion, Chinese Academy of Medical Sciences and Peking Union Medical College (CAMS & PUMC). Briefly, undifferentiated H1 hESCs were seeded on irradiated AGM-S3 cells, cultured in hPSC maintenance medium for 3 days, and then switched to hematopoiesis-inducing medium (referred to as Day 0 [D0]). The co-cultured cells were dissociated with 0.05%–0.25% trypsin/EDTA (ethylenediaminetetraacetic acid) solution (Invitrogen, USA) at the indicated times after D0, and then subjected to FACS or other manipulations.

### 4.3 Monitoring the antagonistic effects of NF-κB signaling inhibitor on *HOXC4* overexpression from D10

D10-induced *HOXC4*/hESCs co-cultured with AGM-S3 cells were treated with 20 nM QNZ (Selleck, USA) starting on D10, and then subjected to analysis of qRT-PCR, FACS, and cell-cycle status at D14. Untreated D10-induced or non-induced cells were used as controls. The qPCR primers are listed in Table S1.

### 4.4 Knockdown of *NF-κB1*

Two *NF-κB1* siRNAs were purchased from Sangon Biotech, China, which detail information of RNA sequences were listed in Table S3. Both of them were proved to efficiently knockdown the expression of *NF-κB1* in 293T cells. Then co-cultures cells were transfected with both two *NF-κB1* siRNAs using Lipofectamine RNAi MAX kit (Invitrogen, USA) from D10, and replaced medium every day. The co-culture cells treated with *NF-κB1* siRNAs or control RNA were subjected to analysis of qRT-PCR, FACS, and cell-cycle status at D14. The qPCR primers are listed in Table S1.

### 4.5 Cell-cycle analysis

*HOXC4/*hESC co-cultures at D14 were treated with 10 μM BrdU for 12 hours, dissociated by treatment with 0.25% trypsin solution, stained with anti-CD43 antibody, and then subjected to analysis of cell-cycle status using the APC-BrdU Flow Kit (BD Biosciences, USA), which was visualized by FACS analysis.

### 4.6 Flow cytometry and cell sorting

Co-cultured cells were dissociated with 0.25% trypsin-EDTA solution (Invitrogen, USA), filtered through a 40 μm nylon mesh to obtain a single-cell suspension, stained with the corresponding antibodies, and then subjected to flow cytometry on a FACSCanto II system or cell sorting on a FACSJazz Sorter (BD Biosciences, USA). All FACS data were analyzed using FlowJo 10. The antibodies used in FACS analyses are listed in Table S2.

### 4.7 Colony culture assay

The hematopoietic potentials of D10-induced or non-induced *HOXC4*/hESC co-culture cells were assessed by culture on methylcellulose (Cat# H4320; STEMCELL Technologies, Canada) supplemented with 100 ng/ml stem cell factor (SCF), 100 ng/ml interleukin-6 (IL-6), 10 ng/ml interleukin-3 (IL-3), 10 ng/ml Fms-related tyrosine kinase 3 ligand (FL), 10 ng/ml thrombopoietin (TPO), 10 ng/ml granulocyte–macrophage colony-stimulating factor (GM-CSF), 4 units/ml erythropoietin (EPO), and 1% penicillin/streptomycin, and then incubated in 5% CO_2_ at 37°C for 14 days. CFU-E was calculated at 7–10 days, and BFU-E, CFU-Mix, and CFU-GM were calculated at 14 days.

### 4.8 Myeloid/erythroid/megakaryocyte differentiation

Non-induced *HOXC4*/hESC co-cultures at D14 were dissociated by treatment with 0.25% trypsin solution and stained with anti-CD34/CD43 antibodies. CD34+CD43+, CD34−CD43^low^, and CD34−CD43^high^ populations were sorted from the corresponding co-culture cells. Next, ∼5 × 10^3^ sorted cells of each type were re-suspended in 250 μl myeloid expansion medium (Cat# 02693, STEMCELL Technologies, Canada), megakaryocyte expansion medium (Cat# 02696, STEMCELL Technologies, Canada), or erythroid expansion medium (containing 10^−6^ M Dexamethasone, 4 U/ml EPO, 100 ng/ml IL-6, and 100 ng/ml SCF), and seeded in 48-well plates. The cells were then cultured with or without DOX induction for 14, 7, or 14 days, respectively, with medium replaced every other day, and then were finally subjected to FACS analysis.

### 4.9 Statistical analysis

All data are presented as means ± SD; statistical analyses were performed using Student's *t* test. *P* < .05 was considered significant.

## Supplementary Material

Supplemental Digital Content

## Supplementary Material

Supplemental Digital Content

## Supplementary Material

Supplemental Digital Content

## Supplementary Material

Supplemental Digital Content

## Supplementary Material

Supplemental Digital Content

## Supplementary Material

Supplemental Digital Content

## Supplementary Material

Supplemental Digital Content

## Supplementary Material

Supplemental Digital Content

## Supplementary Material

Supplemental Digital Content

## References

[1] Amores A Force A Yan Y Zebrafish hox clusters and vertebrate genome evolution. *Science* 1998;282 (5394):1711–1714. 9831563 10.1126/science.282.5394.1711

[2] Rice KL Licht JD. HOX deregulation in acute myeloid leukemia. *J Clin Invest* 2007;117 (4):865–868. 17404613 10.1172/JCI31861 PMC1838955

[3] He H Hua X Yan J. Epigenetic regulations in hematopoietic Hox code. *Oncogene* 2011;30 (4):379–388. 20972460 10.1038/onc.2010.484

[4] Bijl JJ Rieger E van Oostveen JW Walboomers JMM Meijer CJLM. HOXC4, HOXC5, and HOXC6 expression in primary cutaneous lymphoid lesions: High expression of HOXC5 in anaplastic large-cell lymphomas. *Am J Pathol* 1997;151 (4):1067–1074. 9327740 PMC1858029

[5] Meazza R Faiella A Corsetti MT Expression of HOXC4 homeoprotein in the nucleus of activated human lymphocytes. *Blood* 1995;85 (8):2084–2090. 7718879

[6] Auvray C Delahaye A Pflumio F HOXC4 homeoprotein efficiently expands human hematopoietic stem cells and triggers similar molecular alterations as HOXB4. *Haematologica* 2012;97 (2):168–178. 22298821 10.3324/haematol.2011.051235 PMC3269473

[7] Daga A Podesta M Capra MC The retroviral transduction of HOXC4 into human CD34+ cells induces an in vitro expansion of clonogenic and early progenitors. *Exp Hematol* 2000;28 (5):569–574. 10812247 10.1016/s0301-472x(00)00135-1

[8] Xin C Zhao C Yin X Wu S Su Z. Bioinformatics analysis of molecular mechanism of the expansion of hematopoietic stem cell transduced by HOXB4/HOXC4. *Hematology* 2016;21 (8):462–469. 26923762 10.1080/10245332.2015.1101978

[9] Chen B Teng J Liu H Inducible overexpression of RUNX1b/c in human embryonic stem cells blocks early hematopoiesis from mesoderm. *J Mol Cell Biol* 2017;9 (4):262–273. 28992293 10.1093/jmcb/mjx032

[10] Zhou Y Zhang Y Chen B Overexpression of GATA2 enhances development and maintenance of human embryonic stem cell-derived hematopoietic stem cell-like progenitors. *Stem Cell Rep* 2019;13 (1):31–47. 10.1016/j.stemcr.2019.05.007 PMC6626852 31178416

[11] Ran D Shia W Lo M RUNX1a enhances hematopoietic lineage commitment from human embryonic stem cells and inducible pluripotent stem cells. *Blood* 2013;121 (15):2882–2890. 23372166 10.1182/blood-2012-08-451641 PMC3624936

[12] Sun W Teng J Zeng J The piggyBac-based double-inducible binary vector system: a novel universal platform for studying gene functions and interactions. *Plasmid* 2019;105:102420–102425. 31265838 10.1016/j.plasmid.2019.102420

[13] Chang J Sun W Zeng J Establishment of an in vitro system based on AGM-S3 co-culture for screening traditional herbal medicines that stimulate hematopoiesis. *J Ethnopharmacol* 2019;240:111938–111944. 31077780 10.1016/j.jep.2019.111938

[14] Dzierzak E Bigas A. Blood development: hematopoietic stem cell dependence and independence. *Cell Stem Cell* 2018;22 (5):639–651. 29727679 10.1016/j.stem.2018.04.015

[15] Nandakumar SK Ulirsch JC Sankaran VG. Advances in understanding erythropoiesis: evolving perspectives. *Brit J Haematol* 2016;173 (2):206–218. 26846448 10.1111/bjh.13938 PMC4833665

[16] An X Schulz VP Mohandas N Gallagher PG. Human and murine erythropoiesis. *Curr Opin Hematol* 2015;22 (3):206–211. 25719574 10.1097/MOH.0000000000000134 PMC4401149

[17] Choi K Vodyanik MA Slukvin II. Generation of mature human myelomonocytic cells through expansion and differentiation of pluripotent stem cell–derived lin–CD34+CD43+CD45+ progenitors. *J Clin Invest* 2009;119 (9):2818–2829. 19726877 10.1172/JCI38591 PMC2735935

[18] Kawagoe H Humphries RK Blair A Sutherland HJ Hogge DE. Expression of HOX genes, HOX cofactors, and MLL in phenotypically and functionally defined subpopulations of leukemic and normal human hematopoietic cells. *Leukemia* 1999;13 (5):687–698. 10374871 10.1038/sj.leu.2401410

[19] Fongang B Kong F Negi S Braun W Kudlicki A. A conserved structural signature of the homeobox coding DNA in HOX genes. *Sci Rep* 2016;6 (1):35415–35425. 27739488 10.1038/srep35415 PMC5064350

[20] Rezsohazy R Saurin AJ Maurel-Zaffran C Graba Y. Cellular and molecular insights into Hox protein action. *Development* 2015;142 (7):1212–1227. 25804734 10.1242/dev.109785

[21] Rubin MR Toth LE Patel MD D’Eustachio P Nguyen-Huu MC. A mouse homeo box gene is expressed in spermatocytes and embryos. *Science* 1986;233 (4764):663–667. 3726554 10.1126/science.3726554

[22] Featherstone MS Baron A Gaunt SJ Mattei MG Duboule D. Hox-5.1 defines a homeobox-containing gene locus on mouse chromosome 2. *Proc Natl Acad Sci USA* 1988;85 (13):4760–4764. 2898782 10.1073/pnas.85.13.4760 PMC280515

[23] Geada AM Gaunt SJ Azzawi M Sequence and embryonic expression of the murine Hox-3.5 gene. *Development* 1992;116 (2):497–506. 1363091 10.1242/dev.116.2.497

[24] Sauvageau G Thorsteinsdottir U Eaves CJ Overexpression of HOXB4 in hematopoietic cells causes the selective expansion of more primitive populations in vitro and in vivo. *Gene Dev* 1995;9 (14):1753–1765. 7622039 10.1101/gad.9.14.1753

[25] Kyba M Perlingeiro RCR Daley GQ. HoxB4 confers definitive lymphoid-myeloid engraftment potential on embryonic stem cell and yolk sac hematopoietic progenitors. *Cell* 2002;109 (1):29–37. 11955444 10.1016/s0092-8674(02)00680-3

[26] Wang L Menendez P Shojaei F Generation of hematopoietic repopulating cells from human embryonic stem cells independent of ectopic HOXB4 expression. *J Exp Med* 2005;201 (10):1603–1614. 15883170 10.1084/jem.20041888 PMC2212922

[27] Kessel KU Bluemke A Schöler HR Emergence of CD43-expressing hematopoietic progenitors from human induced pluripotent stem cells. *Transfus Med Hemoth* 2017;44 (3):143–150. 10.1159/000477357 PMC5473062 28626365

[28] Bazil V Brandt JE Hoffman R. Resistance of human hematopoietic stem cells to a monoclonal antibody recognizing CD43. *Stem Cells* 1997;15 (S2):13–19. 9368320 10.1002/stem.5530150804

[29] Bravo-Adame ME Vera-Estrella R Barkla BJ An alternative mode of CD43 signal transduction activates pro-survival pathways of T lymphocytes. *Immunology* 2016;150 (1):87–99. 27606486 10.1111/imm.12670 PMC5341503

[30] Choi K Vodyanik MA Togarrati PP Identification of the hemogenic endothelial progenitor and its direct precursor in human pluripotent stem cell differentiation cultures. *Cell Rep* 2012;2 (3):553–567. 22981233 10.1016/j.celrep.2012.08.002 PMC3462245

[31] Vodyanik M Slukvin II Choi K. Hematopoietic differentiation and production of mature myeloid cells from human pluripotent stem cells. *Nat Protoc* 2011;6 (3):296–313. 21372811 10.1038/nprot.2010.184 PMC3066067

[32] Vodyanik MA Thomson JA Slukvin II. Leukosialin (CD43) defines hematopoietic progenitors in human embryonic stem cell differentiation cultures. *Blood* 2006;108 (6):2095–2105. 16757688 10.1182/blood-2006-02-003327 PMC1895535

[33] Wang H Liu Z Li C High-level protein production in erythroid cells derived from in vivo transduced hematopoietic stem cells. *Blood Adv* 2019;3 (19):2883–2894. 31585952 10.1182/bloodadvances.2019000706 PMC6784527

[34] Psaila B Mead AJ. Single-cell approaches reveal novel cellular pathways for megakaryocyte and erythroid differentiation. *Blood* 2019;133 (13):1427–1435. 30728145 10.1182/blood-2018-11-835371 PMC6443046

[35] Li W Wang Y Zhao H Identification and transcriptome analysis of erythroblastic island macrophages. *Blood* 2019;134 (5):480–491. 31101625 10.1182/blood.2019000430 PMC6676133

[36] Palis J. Hematopoietic stem cell-independent hematopoiesis: emergence of erythroid, megakaryocyte, and myeloid potential in the mammalian embryo. *FEBS Lett* 2016;590 (22):3965–3974. 27790707 10.1002/1873-3468.12459

[37] Paluru P Hudock KM Cheng X The negative impact of Wnt signaling on megakaryocyte and primitive erythroid progenitors derived from human embryonic stem cells. *Stem Cell Res* 2014;12 (2):441–451. 24412757 10.1016/j.scr.2013.12.003 PMC4048963

[38] Zeng Y He J Bai Z Tracing the first hematopoietic stem cell generation in human embryo by single-cell RNA sequencing. *Cell Res* 2019;29 (11):881–894. 31501518 10.1038/s41422-019-0228-6 PMC6888893

[39] Mayani H. The regulation of hematopoietic stem cell populations. *F1000Research* 2016;5:1524–1529. 10.12688/f1000research.8532.1 PMC4926728 27408695

[40] Notta F Doulatov S Laurenti E Isolation of single human hematopoietic stem cells capable of long-term multilineage engraftment. *Science* 2011;333 (6039):218–221. 21737740 10.1126/science.1201219

[41] Bereshchenko O Mancini E Moore S Bilbao D Nerlov C. Hematopoietic stem cell expansion precedes the generation of committed myeloid leukemia-initiating cells in C/EBPα mutant AML. *Cancer Cell* 2009;16 (5):390–400. 19878871 10.1016/j.ccr.2009.09.036

[42] Ivanovs A Rybtsov S Anderson RA Medvinsky A. CD43 but Not CD41 marks the first hematopoietic stem cells in the human embryo. *Blood* 2014;124 (21):4330–14330.

[43] McKenzie JL Takenaka K Gan OI Doedens M Dick JE. Low rhodamine 123 retention identifies long-term human hematopoietic stem cells within the Lin-CD34+CD38- population. *Blood* 2007;109 (2):543–545. 16990597 10.1182/blood-2006-06-030270

[44] Notta F Doulatov S Dick JE. Engraftment of human hematopoietic stem cells is more efficient in female NOD/SCID/IL-2Rgc-null recipients. *Blood* 2010;115 (18):3704–3707. 20207983 10.1182/blood-2009-10-249326

[45] Park S Kim P Lee K APRIL stimulates NF-κB-mediated HoxC4 induction for AID expression in mouse B cells. *Cytokine* 2013;61 (2):608–613. 23178148 10.1016/j.cyto.2012.10.018 PMC3723325

