# Supplementary material for: HOXC4 up-regulates NF-κB signaling and promotes the cell proliferation to drive development of human hematopoiesis, especially CD43+ cells
Source: Blood Sci. 2020 Sep 1;2(4):117–28. doi: 10.1097/BS9.0000000000000054 (PMC8974941; doi:10.1097/BS9.0000000000000054)

**Supplemental Figure 1.** Co-cultured non-transgenic H1 hESCs were collected at D2, D4, D6, D8, D10, D12, or D14, and mRNA expression of *HOXA4*, *HOXB4*, *HOXC4*, and *HOXD4* was measured by qRT-PCR. *GAPDH* was used as an internal control.


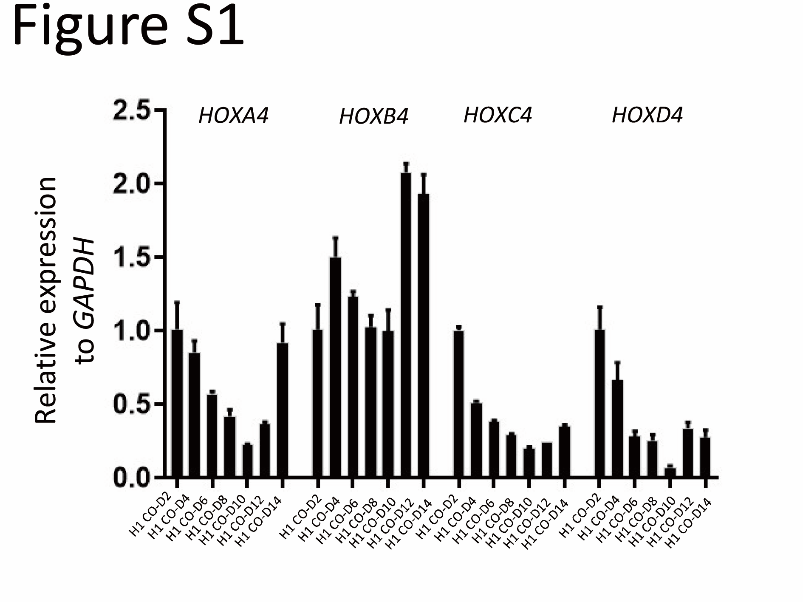

Supplement: Supplemental Digital Content [file bls-2-117-s001.doc]
