# Supplementary material for: HOXC4 up-regulates NF-κB signaling and promotes the cell proliferation to drive development of human hematopoiesis, especially CD43+ cells
Source: Blood Sci. 2020 Sep 1;2(4):117–28. doi: 10.1097/BS9.0000000000000054 (PMC8974941; doi:10.1097/BS9.0000000000000054)

**Supplemental Figure 2.** (A) Amino acid sequence alignment of human *HOXA4*, *HOXB4*, *HOXC4*, and *HOXD4* for conservation analysis of *HOX* gene homologs. Red boxes denote the Homeobox domains of the HOX proteins*.* (B) Amino acid sequence alignment of human, mouse, and zebrafish *HOXC4* genes*.* Red boxes denote the Homeobox domains.


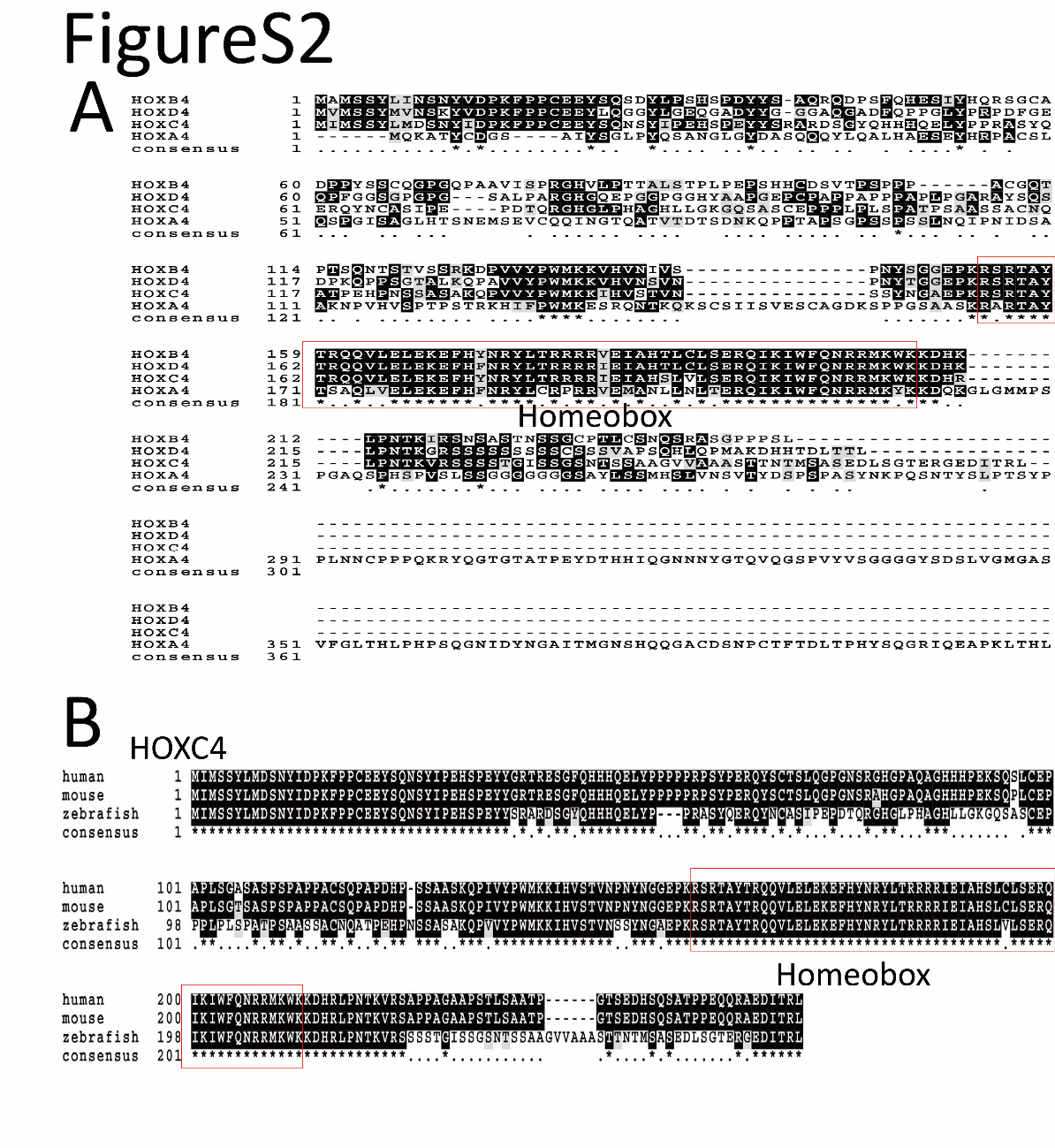

Supplement: Supplemental Digital Content [file bls-2-117-s002.doc]
