# Supplementary material for: HOXC4 up-regulates NF-κB signaling and promotes the cell proliferation to drive development of human hematopoiesis, especially CD43+ cells
Source: Blood Sci. 2020 Sep 1;2(4):117–28. doi: 10.1097/BS9.0000000000000054 (PMC8974941; doi:10.1097/BS9.0000000000000054)

**Supplemental Figure 3.** The overexpression of *HOXC4* from D6 or later promoted hematopoiesis. *HOXC4*/hESCs co-cultured with AGM-S3 cells were treated with DOX from D0, D2, D4, D6, D8, D10, or D12 , and were subjected to FACS analysis using the indicated combination of antibodies against (A) KDR/CD34 (at D4), (B) CD34/CD43 (at D8), and (C) GPA/CD71, CD34/CD43, CD34/CD45 (at D14). The ratios were compared between non-induced co-cultures and the GFP+ fraction of co-cultures treated with DOX from D0, D2, D4, D6, D8, D10, or D12.


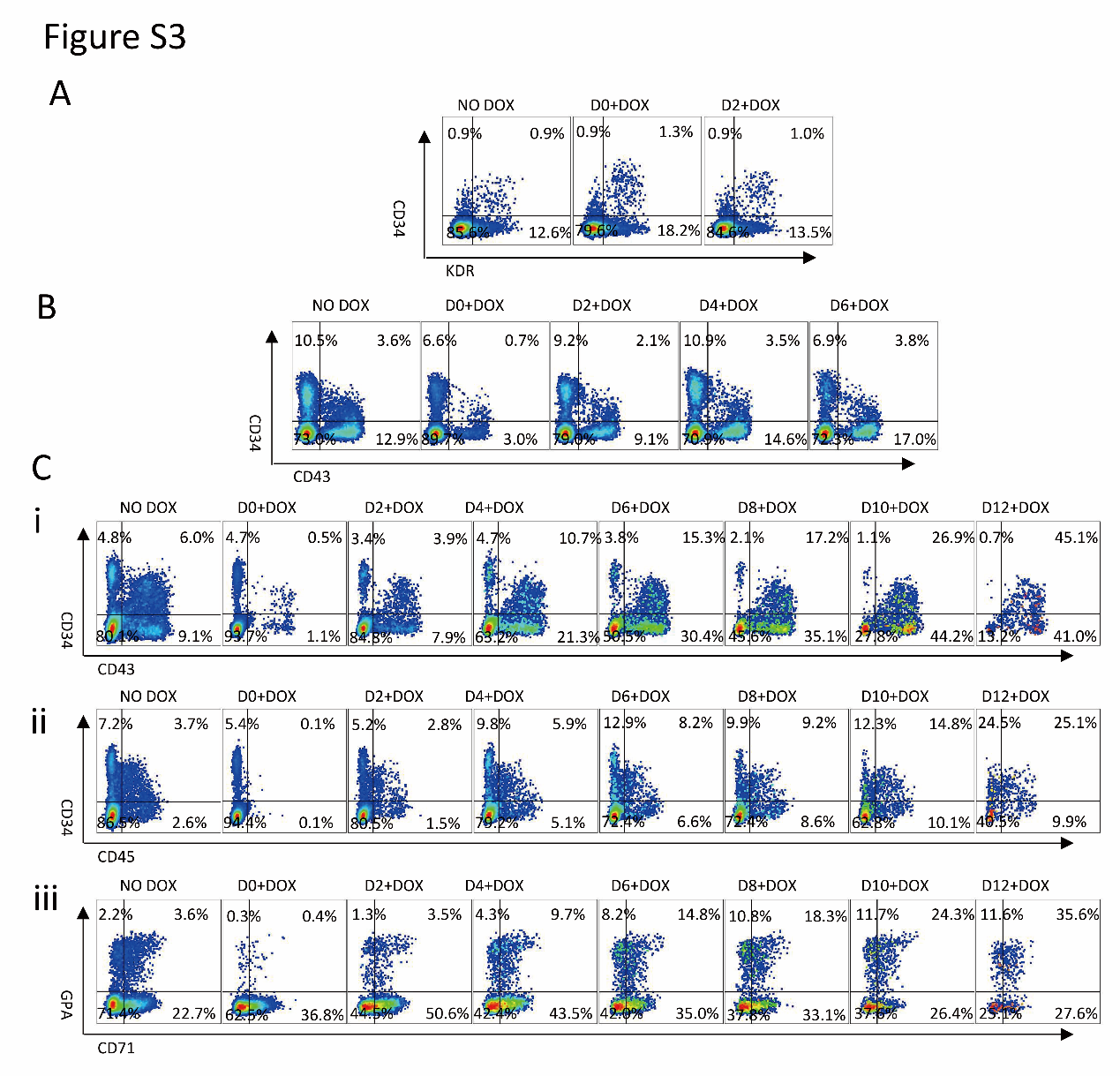

Supplement: Supplemental Digital Content [file bls-2-117-s003.doc]
