# Supplementary material for: HOXC4 up-regulates NF-κB signaling and promotes the cell proliferation to drive development of human hematopoiesis, especially CD43+ cells
Source: Blood Sci. 2020 Sep 1;2(4):117–28. doi: 10.1097/BS9.0000000000000054 (PMC8974941; doi:10.1097/BS9.0000000000000054)

**Supplemental Figure 4.** *HOXC4*/hESCs co-cultured with AGM-S3 induced from D10 were treated with 20 nM QNZ or *NF-kB1* siRNA at the same time, then subjected to FACS analysis at D14, using the indicated antibody combinations against CD34/CD43, CD34/CD45, or GPA/CD71.


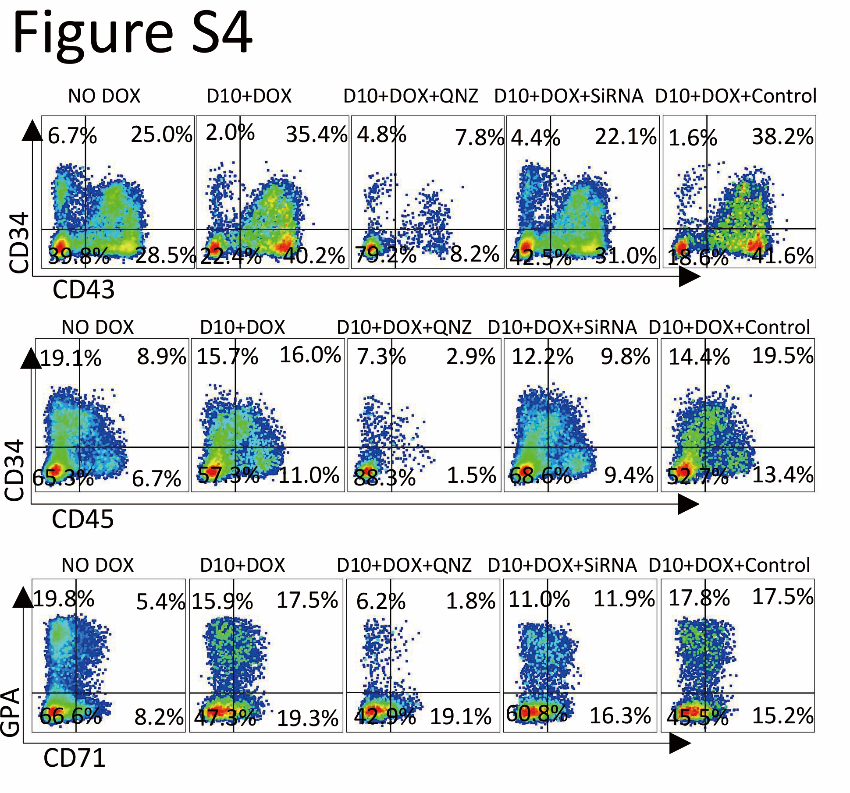

Supplement: Supplemental Digital Content [file bls-2-117-s004.doc]
