# Supplementary material for: HOXC4 up-regulates NF-κB signaling and promotes the cell proliferation to drive development of human hematopoiesis, especially CD43+ cells
Source: Blood Sci. 2020 Sep 1;2(4):117–28. doi: 10.1097/BS9.0000000000000054 (PMC8974941; doi:10.1097/BS9.0000000000000054)

**Supplemental Figure 5.** Co-cultured *HOXC4*/hESCs were induced with DOX from D0, D2, D4, D6, D8, D10, or D12, and the GFP+ fractions of induced co-cultures were analyzed by FACS at D14 using the indicated antibodies combination of CD34/CD43/GPA/CD41a. The results were compared with those from non-induced co-cultures.


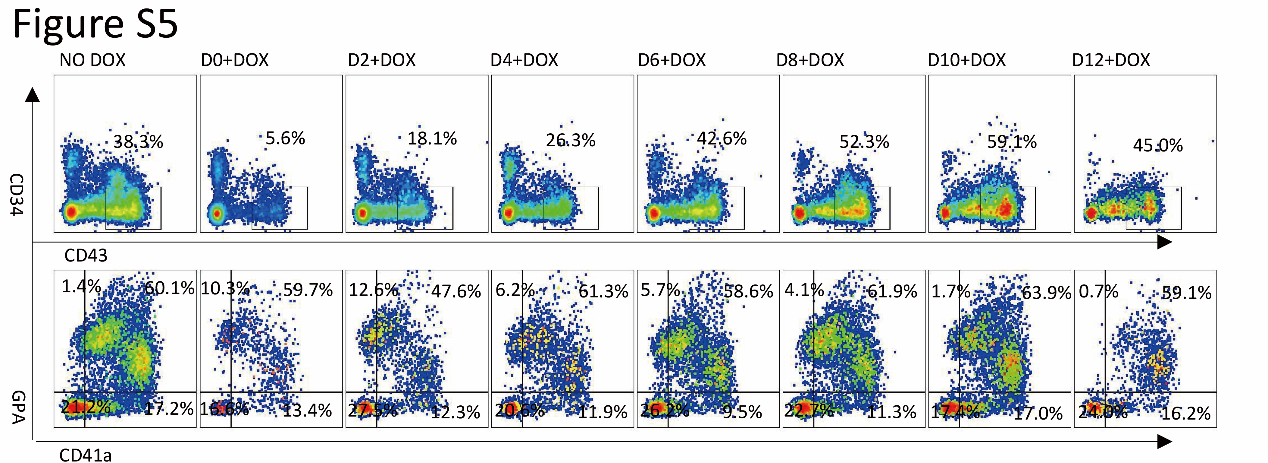

Supplement: Supplemental Digital Content [file bls-2-117-s005.doc]
