# Supplementary material for: HOXC4 up-regulates NF-κB signaling and promotes the cell proliferation to drive development of human hematopoiesis, especially CD43+ cells
Source: Blood Sci. 2020 Sep 1;2(4):117–28. doi: 10.1097/BS9.0000000000000054 (PMC8974941; doi:10.1097/BS9.0000000000000054)
Supplement: Supplemental Digital Content [file bls-2-117-s006.doc]

**TABLE S1**

Primers used for qRT-PCR analysis

| Gene | Forward primer (5′→3′) | Reverse primer (5′→3′) |
| --- | --- | --- |
| *GAPDH* | GTCTCCTCTGACTTCAACAGCG | ACCACCCTGTTGCTGTAGCCAA |
| *NF-KB1* | GAAGAGGAAGAAAATGGTGGAGT | AAACACAGAGGCTGGTTTTGTAA |
| *HOXA4* | TGACCATGAGCTCGTTTTTG | CTCCTTCTCCAGCTCCAAGA |
| *HOXB4* | GCACTGCCTTTTTCTGTTCC | TTCCTTCTCCAGCTCCAAGA |
| *HOXC4* | CCAGCAAGCAACCCATAGTC | GGGTCAGGTAGCGGTTGTAA |
| *HOXD4* | TGGTCTACCCCTGGATGAAG | ACCGGTGTAGTTGGGGTTC |
