# Supplementary material for: HOXC4 up-regulates NF-κB signaling and promotes the cell proliferation to drive development of human hematopoiesis, especially CD43+ cells
Source: Blood Sci. 2020 Sep 1;2(4):117–28. doi: 10.1097/BS9.0000000000000054 (PMC8974941; doi:10.1097/BS9.0000000000000054)
Supplement: Supplemental Digital Content [file bls-2-117-s007.doc]

**TABLE S2**

**Antibodies used for FACS analyses**

| Antibodies | Brand | Antibody channels | Cat |
| --- | --- | --- | --- |
| CD309(KDR) | BD | PE | 560494 |
| CD34 | BD | APC | 555824 |
| CD43 | BD | PE | 560199 |
| CD43 | BD | PE-CY7 | 563522 |
| CD45 | BD | PE-CY7 | 557748 |
| CD235a(GPA) | BD | APC | 551336 |
| CD71 | BD | PE | 561938 |
| 7-AAD | BD | 7-AAD | 559925 |
| CD41a | BD | PE-CY7 | 561424 |
| CD42b | BD | PE | 555473 |
| CD15 | BD | PE-CY7 | 560827 |
