# Supplementary material for: HOXC4 up-regulates NF-κB signaling and promotes the cell proliferation to drive development of human hematopoiesis, especially CD43+ cells
Source: Blood Sci. 2020 Sep 1;2(4):117–28. doi: 10.1097/BS9.0000000000000054 (PMC8974941; doi:10.1097/BS9.0000000000000054)
Supplement: Supplemental Digital Content [file bls-2-117-s008.doc]

**TABLE S3**

**Sequence of *NF-κB1* siRNA**

| Name | sense (5′→3′) | antisense (5′→3′) | |  |
| --- | --- | --- | --- | --- |
| NF-κB1-1 | CGAAUGACAGAGGCGUGUAUATT | | UAUACACGCCUCUGUCAUUCGTT | |
| NF-κB1-2 | CCAGAGUUUACAUCUGAUGAUTT | | AUCAUCAGAUGUAAACUCUGGTT | |
