# Supplementary material for: HOXC4 up-regulates NF-κB signaling and promotes the cell proliferation to drive development of human hematopoiesis, especially CD43+ cells
Source: Blood Sci. 2020 Sep 1;2(4):117–28. doi: 10.1097/BS9.0000000000000054 (PMC8974941; doi:10.1097/BS9.0000000000000054)
Supplement: Supplemental Digital Content [file bls-2-117-s009.doc]

**Supplemental materials and methods**

***Western blotting analysis***

The cells were lysed by gently tapping with RIPA buffer (Sigma,USA). After centrifugation (12,000×g, 4°C, 5 min), the supernatants were transferred to clean tubes, and protein concentrations were measured by the BCA method. Proteins were denatured at 100°C for 5 min and then resolved by denaturing SDS-PAGE (5% separation gel / 10% stacking gel) according to the standard protocol for the Bio-Rad Mini-PROTEAN Tetra Cell electrophoresis system. Bio-Rad Trans-Blot cells were used to transfer proteins to PVDF membranes in 1× transmembrane buffer (3 g Tris, 14.5 g Glycine, 100 ml methanol in 1 L ddH_2_O). Thereafter, the membranes were incubated at 4°C overnight with primary antibodies against SOX2 (1: 1000 dilution; cat#: sc-365823, Santa Cruz Biotechnology, USA ), OCT4 (1: 1000 dilution; cat#: sc-5279, Santa Cruz Biotechnology, USA), NANOG (1: 1000 dilution; cat#: sc-293121, Santa Cruz Biotechnology, USA), HOXC4 (1: 200 dilution; cat#: SC398460, Santa Cruz Biotechnology, USA), or GAPDH (1: 1000 dilution; cat#: KC-5G4, Kang Chen, China) in blocking solution (1× TBST containing 5% skim milk powder). The membranes were washed with 1× TBST, incubated with goat anti-mouse IgG H&L (1: 1000 dilution; cat#: ZB-2305, ZSBIO, China) conjugated with horseradish peroxidase at room temperature for 1 h, and washed again with 1× TBST. The Amersham ECL Prime Immunoblotting detection kit (Cat. No. RPN2232, GE Healthcare, USA) was used to visualize immune-reactive bands. Signals were quantified using the ImageQuant LAS4000 mini system. Molecular weight was determined by referring to a pre-stained protein ladder (cat#: 26626, PageRuler, USA).

***Immunofluorescence staining***

Cells isolated from BFU-E colonies were spun onto slides, fixed with 4% paraformaldehyde (PFA) for 30 min at 4°C, and washed three times with PBS. They were then incubated with membrane permeation reagent (PBS containing 0.1% Triton-100 and 5% skim milk powder) at 4°C for 30 min, washed three times with PBS containing 5% skim milk, stained overnight at 4°C with anti-hemoglobin antibody (goat anti-human, #cat: A80-134A, Bethyl, USA), washed three times with PBS containing 5% skim milk, and then incubated for 30 min at room temperature with secondary antibodies (Cy3-conjugated secondary Ab, Jackson ImmunoResearch, donkey anti-goat, cat#: 705-165-003, USA). Nuclei were labeled with Hoechst 33342 (Molecular Probes). After washing three times with PBS, the sample was imaged under a fluorescence microscope.

***May–Grunwald–Giemsa staining***

Cells from BFU-E colonies were harvested and spun onto glass slides using the Cell Cytospin 4 (Thermo Fisher Scientific), dyed in May–Grunwald solution (MERCK No. 101424, [Germany](javascript:;)) for 10 min, and further stained in Giemsa B solution for 2–3 min. After washing, a mixture of Giemsa A solution (MERCK No. 10924, [Germany](javascript:;)) and Giemsa B solution was added in a 1: 20 ratio, and the sample was incubated for 20 min. After staining, the samples were washed with water and air dried. Cells were imaged under an oil emersion lens on an Olympus BX53 microscope.

***Quantitative reverse transcription PCR (qRT-PCR)***

Total RNA was extracted from 5 × 10^5^ cells using 500 µl TRIzol (Life Technologies, USA). Complementary DNA was synthesized using a reverse transcription kit (Bio-Rad, USA). Each 20 μl reaction contained 4 μl of 5×Master Mix, 1 μl reverse transcriptase, 1 μg total RNA, and nuclease-free water to 20 μl. The program used for reverse transcription was as follows: 25°C for 5 min, 42°C for 30 min, 85°C for 5 min, and hold at 4°C. qPCR was performed using the Fast Start Universal SYBR Green Master (Roche, USA) on a CFX96 real-time system (Bio-Rad, USA). Each 15 µl reaction contained 7.5 μl of 2×Master Mix, 0.4 μl of each primer (10 µM), 4.7 μl H_2_O, and 2 μl complementary DNA. Amplification conditions were set up as follows: denaturation for 10 min at 95°C, followed by 45 cycles of 95°C for 15 sec, 58°C for 30 sec, and 72°C for 30 sec. Glyceraldehyde 3-phosphate dehydrogenase (*GAPDH*) served as an internal control. The qPCR primers are listed in Table S1.
